# Rate of Involved Endocervical Margins According to High-Risk Human Papillomavirus Subtype and Transformation Zone Type in Specimens with Cone Length ≤ 10 mm versus > 10 mm—A Retrospective Analysis

**DOI:** 10.3390/life13081775

**Published:** 2023-08-20

**Authors:** Chiara Paternostro, Elmar A. Joura, Christina Ranftl, Eva-Maria Langthaler, Robin Ristl, Tim Dorittke, Sophie Pils

**Affiliations:** 1 Department of Obstetrics and Gynecology, Medical University of Vienna, 1090 Vienna, Austria; chiara.paternostro@meduniwien.ac.at (C.P.); christina.ranftl@gmx.at (C.R.); tim.dorittke@meduniwien.ac.at (T.D.); sophie.pils@meduniwien.ac.at (S.P.); 2 Department of Pathology, Medical University of Vienna, 1090 Vienna, Austria; eva.langthaler@meduniwien.ac.at; 3 Center of Medical Statistics, Informatics and Intelligent Systems, Medical University of Vienna, 1090 Vienna, Austria; robin.ristl@meduniwien.ac.at

**Keywords:** high-grade cervical intraepithelial neoplasia, conization, adverse pregnancy event, cervical integrity, cervical tissue loss, high-risk HPV, oncological safety, preterm delivery, squamocolumnar junction

## Abstract

The aim of this study was to evaluate the endocervical margin status according to transformation zone (TZ) and high-risk HPV (hr-HPV) subtype in specimens with cone length ≤ 10 mm versus > 10 mm to provide data for informed decision making and patients counseling especially for women wishing to conceive. In this retrospective cohort study, 854 patients who underwent large loop excision of the transformation zone during a nine-year period (2013–2021) for cervical disease were analyzed. The main outcome parameters were excision length, histological result, TZ type, HPV subtype and endocervical margin status. A subgroup analysis was performed according to excision length, with a cut-off value of 10 mm. A two-step surgical procedure was performed in case of an excision length of > 10 mm. The overall rate of positive endocervical margins irrespective of excision length was 17.2%, with 19.3% in specimens with ≤ 10 mm and 15.0% with > 10 mm excision length. Overall, 41.2% of women with a visible TZ and HPV 16/hr infection and 27.0% of women with HPV 18 received an excisional treatment of > 10 mm length without further oncological benefit, respectively. In contrast, assuming that only an excision of ≤ 10 mm length had been performed in women with visible TZ, the rate of clear endocervical margins would have been 63.7% for HPV 16/hr infections and 49.3% for HPV 18 infections. In conclusion, the decision about excision length should be discussed with the patient in terms of oncological safety and the risk of adverse pregnancy events. An excision length > 10 mm increases the number of cases with cervical tissue removed without further oncological benefit, which needs to be taken into account in order to provide an individual therapeutic approach. Furthermore, HPV 18 positivity is related to a higher rate of positive endocervical margins irrespective of TZ.

## 1. Introduction

Cervical intraepithelial neoplasia is associated with a persistent infection with high-risk human papillomavirus, known to be most prevalent during women’s reproductive years [1,2]. Oncological risk varies between high-risk HPV (hr-HPV) genotypes, with HPV 16 and 18 accounting for approximately 70% of invasive cervical cancers [3,4,5]. Large loop excision of the transformation zone (LLETZ) is the most popular method of treatment for high-grade cervical intraepithelial neoplasia (HSIL, formerly CIN 2 and 3) [6,7,8]. Three types of excisional techniques (defined according to the transformation zone (TZ)) were described in 2011 by the International Federation of Cervical Pathology and Colposcopy (IFCPC), with excision type 1 applied for completely visible, ectocervically situated TZ (i.e., TZ 1), excision type 2 for a TZ completely visible after extension (i.e., TZ 2), resecting a small part of endocervical epithelium, and excision type 3 if the TZ is not visible (i.e., TZ 3), including a significant amount of endocervical tissue [9,10]. While it is suggested that an excised cone length (distance between the ectocervical and the endocervical margin) of 10 mm should not be exceeded for a completely visible TZ, a considerably longer excision should be applied to a non-visible TZ [11,12]. Nevertheless, the advised length of cervical excision in relation to the TZ remains a recommendation and cannot be generalized for every patient, since the length of the cervix itself can vary considerably, as well as the location and extent of the neoplastic lesion. Thus, the appropriate application of excisional treatment in order to remove as little cervical tissue as possible but as much as necessary in patients of childbearing age wishing to conceive is essential, given that an excision length > 10 mm has been described as an independent risk factor for prematurity and premature rupture of membranes, and should be determined individually [13,14,15]. In addition, adverse pregnancy events due to cervical incompetence after LLETZ have been reported in several studies and meta-analyses with a directly correlated risk of preterm birth to the dimensions of the cone, which particularly increases with an augmenting cone length [13,16,17,18]. On the other hand, endocervical margin positivity is a predictor for therapeutic failure, i.e., persistent or recurrent HSIL, occurring in up to 25% of cases, which is associated with a higher rate of hr-HPV persistence post-LLETZ [19,20,21,22]. Therefore, it is crucial for individual management to weigh up the risk of undertreatment, with subsequent measures and even potential progression to cervical cancer, against the occurrence of adverse pregnancy events. The aim of this study is to evaluate the endocervical margin status according to TZ and hr-HPV subtype comparing specimens with ≤ 10 mm versus > 10 mm cone length as well as subsequently provide data for informed decision making and patient counseling, especially for women wishing to conceive. 

## 2. Materials and Methods

In this retrospective single-center cohort study, we identified 1035 patients who underwent LLETZ for cervical neoplasia between 2013 and 2021, of whom 181 patients had to be excluded, resulting in 854 cases eligible for analysis. Inclusion criteria were as follows: (i) preoperative colposcopy with documentation of the TZ; (ii) preoperative analysis of the hr-HPV status; (iii) LLETZ at the Department of Obstetrics and Gynecology, Medical University of Vienna; (iv) measurement of cone dimensions. Exclusion criteria were as follows: (i) ≤18 years of age; (ii) LLETZ before January 2013 or after December 2021; (iii) pregnancy. Cervical cytology via PAP smear as well as a colposcopy including a biopsy was conducted prior to excisional treatment. LLETZ was performed in the operating room, and the surgical specimen was marked by the surgeon at 12 o’clock. All specimens were reviewed and measured by gynecologic pathologists. All three dimensions of the cone were assessed in millimeters, i.e., anteroposterior dimension, transverse dimension and length. TZ was documented in the medical records either as “visible/non-visible” or specified by the three subgroups “TZ 1/2/3”. Hence, in order to perform sub-analysis, TZ type 1 (squamocolumnar junction completely visible) and type 2 (i.e., squamocolumnar junction partially visible) were merged into the subgroup “fully visible TZ”, and type 3 (squamocolumnar junction not visible) was denoted “non-visible TZ” [9,10]. A cone resection of ≤ 10 mm of length was performed by removing one specimen with a large loop in a standard fashion. In case of an excision length of > 10 mm, a two-step procedure was performed by first removing a specimen of the ectocervically situated TZ (not more than 10 mm of length) followed by an additional smaller loop to remove the endocervical part of the TZ, resulting in a total length of both specimens of more than 10 mm. The type of excision was applied according to the surgeons’ clinical assessment due to the TZ type and expansion of the lesion or upon informed patients’ request, preferring oncological safety rather than low risk of preterm delivery. Since an excisional treatment of removing more than 10 mm cervical tissue is known to be an independent risk factor for adverse pregnancy events, we chose to compare two subgroups with a cut-off value of 10 mm to provide data for informed patient education, particularly for women of childbearing age with a desire to get pregnant [15]. Given the fact that an involved endocervical margin is especially known to be a predictor for treatment failure, i.e., persistent disease, we chose to evaluate only the endocervical margin status [12,22]. Endocervical margin positivity was referred to histological confirmation of HSIL or carcinoma on the surgical margin and designated as “involved” or “clear”. Hr-HPV genotypes were diagnosed using the “cobas HPV test” (Roche Diagnostics, Indianapolis, Indiana) and described individually (HPV 16 and 18) or in a pooled category reported as other high-risk HPV (HPV 31, 33, 35, 39, 45, 51, 52, 56, 58, 59, 66 and 68). For sub-analyses, hr-HPV types were grouped according to specific oncological characteristics. HPV 18 is more likely to cause potentially endocervical adenocarcinoma in situ (AIS), while HPV 16 and other hr-HPV (“HPV 16/hr”) types are predominantly causing squamous cell carcinoma (SCC) [23,24]. Clinical and pathological data were extracted retrospectively from medical records. The main outcome measures were excision length, TZ type, hr-HPV status and endocervical margin status. In addition, the following clinical parameters were included: age of the patient at LLETZ, cervical cytology diagnosed by a PAP smear, histological result of colposcopic biopsy and LLETZ specimen. Nominal variables are reported as numbers and frequencies, and continuous variables are reported as medians and interquartile ranges (IQRs). Statistical analyses were performed with SPSS v26 (released 2019, IBM Corp., Armonk, NY, USA) using the Mann–Whitney U test or the Pearson’s chi-square test, where appropriate. A univariate analysis was performed in order to evaluate the effect of an excision length > 10 mm on the endocervical resection margin of the specimen. A two-sided *p* value < 0.05 was considered significant. Figures were generated using Microsoft Word (version 16.43) and SankeyMATIC software.

## 3. Results

A total of 854 patients were included in the analysis with a median age of 36 years (IQR 31–44; Table 1). The accordance rate of the histological result between cervical cytology and LLETZ specimen was 62.5% (n = 498/797) for LSIL/HSIL and PAP III/IIID/IV, 54.2% (n = 13/24) for AIS and PAP IIIG/IV, and 12.1% (n = 4/33) for SCC/AC and PAP IV/V. Furthermore, the rate of consistent histological results between preoperative colposcopic biopsy and LLETZ specimen was the highest for HSIL (76.6%), followed by AIS (33.3%), LSIL (31.5%) and SCC/AC (9.1%). Women receiving an LLETZ with an excision length ≤ 10 mm were significantly younger than women with an excised cone length > 10 mm (34 years (IQR 30–41) vs. 38 years (IQR 32–46), *p* < 0.001; Table 1). Clear endocervical margins were obtained in a total of 82.8% of the patients with no significant difference between specimens with a length ≤ 10 mm versus > 10 mm. The majority of the cases had a visible TZ (n = 701/854; Table 1), and HSIL was found most often in the histological examination of the LLETZ specimen (n = 673/854, Table 1). The rate of specimens with an excision length of ≤ 10 mm versus > 10 mm of cases with HPV 18 compared to HPV 16/hr did not significantly differ (Table 1). Basic characteristics are shown in Table 1. 

**Figure 1:**
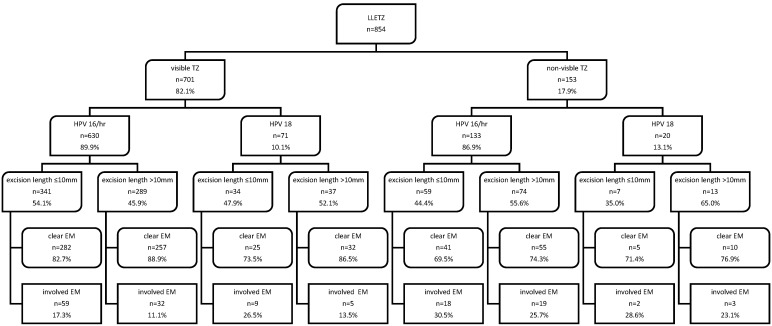
Endocervical margin status in specimens with a cone length ≤ 10 mm versus > 10 mm grouped by TZ and hr-HPV type. Data are presented as number and frequencies. Abbreviations used are as follows: LLETZ, large loop excision of the transformation zone; TZ, transformation zone; HPV, human papillomavirus; EM, endocervical margin.

Specimens with HPV 18 received a higher median excision length (12 mm, IQR 9–15 mm) than cases with HPV 16/hr (10 mm, IQR 7–15 mm; *p* = 0.078). AIS was diagnosed significantly more often in patients with HPV 18 than HPV 16/hr (12.1% versus 1.8%; *p* < 0.001), whereas HSIL was detected significantly more frequently in patients with HPV 16/hr (85.3% versus 68.7%; *p* < 0.001). In addition, HPV 18 caused 11 cases of AIS, which were detected correctly via a preoperative biopsy in 4 cases (36.4%). Women with a visible TZ were significantly younger than those with a non-visible TZ (35 years (IQR 30–41 years) vs. 48 years (IQR 38–55 years), respectively; *p* < 0.001). The distribution of clear and involved endocervical margins according to the TZ and hr-HPV subtype with regard to the 10 mm cut-off of the excision length is illustrated in Figure 1. Within the analyzed subgroups, the rate of clear endocervical margins is higher in case of an excision length > 10 mm versus ≤ 10 mm. Furthermore, women with a visible TZ obtained a complete endocervical resection of neoplasia significantly more often (85.7% vs. 72.5%, *p* < 0.001). The lowest rate of clear endocervical margins was observed in women with a non-visible TZ and an excision length of < 10 mm (69.5% with HPV 16/hr and 71.4% with HPV 18, Figure 1.

The effect of an excision > 10 mm of length on the endocervical margin status is presented in Table 2. A lower odds ratio to obtain a clear endocervical margin for patients receiving an excision ≤ 10 mm of length was found within all analyzed subgroups. A statistically significant effect on endocervical margin negativity was found only in women with a visible TZ and a concomitant HPV 16/hr infection, which were found to have a 1.68 higher chance to obtain a negative endocervical margin with an excision length > 10 mm (OR = 1.68 (CI 1.06–2.67), *p* = 0.027; Table 2). 

**Figure 2:**
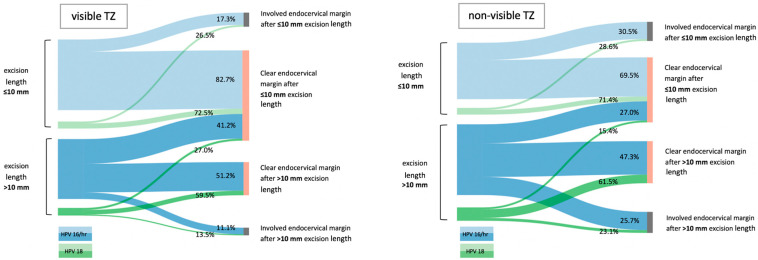
Illustration of the potential patients’ benefit of an excision length ≤ 10 mm in case of a longer resection of > 10 mm. Abbreviations used are as follows: TZ, transformation zone; HPV, human papillomavirus.

The two-step surgical technique in specimens of more than 10 mm of length enabled the creation of a flow diagram for each TZ type subgroup (visible TZ vs. non-visible TZ; Figure 2), illustrating the potential benefit to patients of an excision length ≤ 10 mm in case of conducting a longer excision. In the analyzed collective, 41.2% of women with a visible TZ and HPV 16/hr and 27.0% with a visible TZ and HPV 18 received an excisional treatment > 10 mm of length without further oncological benefit. In patients with a non-visible TZ, this rate was found to be 27.0% for HPV 16/hr and 15.4% for HPV 18, respectively. 

On the other hand, assuming that only an excision with ≤ 10 mm length had been performed, the rate of clear endocervical margin status would have been 63.7% (n = 401/630) for HPV 16/hr infections and 49.3% (n = 35/71) for HPV 18 infections in visible TZ and 45.9% (n = 61/133) for HPV 16/hr infections and 35.0% (n = 7/20) for HPV 18 infections (Figure 2). In contrast, the actual overall rate of clear endocervical margin status irrespective of excision length was 82.8% (n = 707/854) (Table 1).

## 4. Discussion

In this single center retrospective study, we evaluated the endocervical margin status of 854 LLETZ specimens, resulting in a total rate of 82.8% clear endocervical margins. The rate of involved endocervical margins in a cone excision of ≤ 10 mm length was observed to increase with a non-visible versus visible TZ and with a concomitant HPV 18 vs. HPV 16/hr infection (Figure 1). Even though the chance of obtaining a clear endocervical margin is 1.68 higher with an excision length > 10 mm in the subgroup of women with a visible TZ and HPV 16/hr (OR = 1.68 (CI 1.06–2.67), *p* = 0.027; Table 2), the percentage of cases with cervical tissue removed without further oncological benefit was found to be highest in patients within this subgroup (41.2%, Figure 2). Due to the younger age of women in this group, the increased risk of adverse pregnancy events like preterm delivery or premature rupture of membranes may be a primary concern in patient counseling [14]. 

In case of an excision length ≤ 10 mm, endocervical margin negativity was achieved in 80.7% and in 85.0% in specimens >10 mm length, regardless of TZ or hr-HPV subtype. Given that a clear margin resection rate of > 80% is acknowledged as a quality indicator, specimens with a length ≤ 10 mm and >10 mm would meet the demanded criteria [25,26]. Nevertheless, a considerable dissimilarity within the subgroups, according to TZ and hr-HPV subtype, was observed. While the advised clear margin cut-off level of > 80% in specimens with a length ≤ 10 mm was found in women with a visible TZ and an HPV 16/hr infection, the rate of cases with a visible TZ and an HPV 18 infection reached the 80% threshold only in the event of an excised length > 10 mm (Table 1). It is noteworthy that in the studied patient collective, the total rate of clear endocervical margins in women with a non-visible TZ was only 72.5%, with rates in the range of 69.5–76.9% within the subgroups (Figure 1), which is comparable to published data [27,28].

While several studies have been published evaluating a cut-off level for cone length to prevent involved margins, the reported measures diverge in a wide range between 9 mm and 20 mm, indicating a lack of consensus of an optimal excision length [18,20,29,30,31]. Although recommendations about the optimal excision length according to the TZ type have been published, the ideal cone length needs to be evaluated on an individual basis, especially taking into account anatomical differences in the cervix as well as the extent and number of cervical quadrants covered by the neoplastic lesion [8,29]. Several variables associated with positive endocervical margins have been examined, such as a 34.2-times higher chance for involved endocervical margins in case of more than two cervical quadrants covered by the neoplastic lesion, as described by Giannella L. et al. [29]. Furthermore, Giannini A. et al. showed that positive endocervical rather than ectocervical margins are associated with an increased risk of persistence and/or recurrence [22]. Other studies indicate factors like older age, multiparity and disease severity associated with endocervical margin positivity [13,31]. Gertrudes L. et al. found that an excision length between 10 and 15 mm was associated with a significantly higher chance (with a calculated OR of 2.01) of obtaining clear endocervical margins in women with a TZ 1, even though the overall rate of clear endocervical margin was observed in three out of four patients regardless of performed excision length [12]. This aligns with our findings demonstrated in Table 2. Beyond that, our data provide evidence that the hr-HPV subtype should be taken into account in addition to the TZ type, advising a different consultation approach in cases with HPV 16/hr versus HPV 18. 

Although an OR above 1, which indicates a tendency to obtain clear endocervical margins in case of an excision length > 10 mm, was observed in women within all subgroups (Table 2), a statistically significant difference was found only in women with a visible TZ and HPV 16/hr infection, which might be attributed to the considerably lower number of analyzed cases within the other subgroups. 

Even though endocervical margin involvement has been found to be a risk factor for treatment failure, an observational cohort study by Ang C. et al. discovered that the recurrence rate in a subgroup of women ≤ 35 years of age was similar in specimens of < 10 mm versus ≥ 10 mm length despite a higher rate of involved endocervical margins when excising < 10 mm [32]. Therefore, a possible approach in women with a wish to conceive could be to offer an excision length below 10 mm regardless of TZ, with a mandatory HPV test conducted 6 months after LLETZ, specifically considering that post-treatment HPV testing is known to have a higher sensitivity to predict residual or recurrent disease than margin status (91.0% versus 55.8%) [19,33]. It is noteworthy that in women with suspected invasive and/or glandular disease, this approach cannot be advised generally given the indisputable importance for oncological safety in those particular cases.

Assuming that only an excision length ≤ 10 mm would have been performed, the hypothetical rate of clear endocervical margins in the studied patient collective decreases considerably within all the subgroups ranging from 35.0% (non-visible TZ and HPV 18) to 63.7% (visible TZ and HPV 16/hr) in comparison to the actual observed overall rate of 82.8% (Table 1). Hence, a resection length ≤ 10 mm throughout the studied patient cohort will in fact not lead to an achievement of > 80% clear margins, but it can be discussed as a therapeutic approach especially in patients with a visible TZ and HPV 16/hr infection and the desire to get pregnant. Since HPV 18 is associated with (pre)cancerous lesions frequently of glandular origin, which are often situated within the endocervical canal, a higher excision length independent of the TZ seems to be justifiable to ensure treatment effectiveness and is compatible with our findings (Figure 1) [24,34]. 

In case of a non-visible TZ, an excision of only ≤ 10 mm length—as shown in Figure 2—would result in clear endocervical margin rates below 50% with 45.9% (n = 61/133) for HPV 16/hr and 35.0% (n = 7/20) for HPV 18. Given the observed trend toward delayed childbearing beyond age 30 as well as the increasing demand for artificial reproductive technologies, the amount of women with a non-visible TZ who wish to get pregnant and require a LLETZ for cervical neoplasia may be augmenting over the coming years, which is why we consciously chose to include this group in the analysis [35,36,37]. Since colposcopic assessment as well as the collection of cervical biopsies in women with a non-visible TZ is more likely to be compromised, leading to a lower threshold to conduct an excisional treatment, counseling for this particular subgroup can be challenging. 

The provided information of Figure 1 graphically illustrates real world data of a tertiary referral center throughout a period of nine years, which can be used as a tool for patient consultation to promote informed patient decision making according to TZ and high-risk HPV subtype. Although a two-step surgical procedure is not the state of the art anymore, and its routine performance was avoided at our referral center, given that current guidelines and recommendations advise an excision of one single cervical specimen in order to facilitate histological examination, the two-step surgical procedure for specimens >10 mm length in this historical cohort enabled the creation of Figure 2, demonstrating a notional outcome assuming an excision length of ≤ 10 mm only, and its clinical implication, as demonstrated by the rate of excisions without further oncological benefit [7].

The cut-off threshold of ≤ 10 mm versus > 10 mm excision length was determined according to several published studies and meta-analyses indicating an augmenting risk for adverse obstetric events in case of an excision of more than 10 mm, even though the possible obstetric risk due to a cone excision less than 10 mm in length still remains unclear [8,16,38]. Another potential aspect which is worth noting and discussed in the literature is the inflammation process due to a persistent hr-HPV infection causing adverse pregnancy events independent of surgical or conservative treatment strategy for HSIL, which suggests that a multifactorial pathogenesis underlying the associated obstetric morbidity is possible and needs further prospective investigations [39,40].

An additional issue worth mentioning is the lack of consensus in published data for standardized terminology for cone dimensions after local conservative treatment. Despite the fact that the “2011 colposcopic terminology of the International Federation for Cervical Pathology and Colposcopy” was published in 2012, advising the implementation of an unified nomenclature, a wide variety of terms used, especially when describing the distance between the endocervical and the ectocervical margins, can still be observed [9]. The recently published “Terminology for cone dimensions after local conservative treatment for cervical intraepithelial neoplasia and early invasive cervical cancer: 2022 consensus recommendations from ESGO, EFC, IFCPC, and ESP” by Kyrgiou M. et al. once again depicts the importance of an internationally used nomenclature for cone dimension, that particularly suggests to abandon the terms “depth” and “height” but use “length” instead [8]. 

The major strengths of this study are the large number of analyzed cases giving an overview of real-world data of a tertiary referral center and, in particular, the inclusion of hr-HPV status in addition to the excision length and TZ type, which we consider as an important factor contributing to the originality of this analysis. To our knowledge, this is the first study showing the astonishing amount of cervical tissue removed without oncological benefit according to TZ and hr-HPV subtype (Figure 2), which is crucial to enhance the awareness for individual treatment approach especially in patients with a wish to conceive. Due to missing data, it was not possible to analyze TZ type 1 and type 2 separately, which we consider a limitation of this study due to the retrospective character and the broad study period. Other limitations include the low rate of non-visible TZ and HPV 18 infections, unavailable data of singular subtype evaluation within the pooled hr-HPV subtypes like HPV 45, known to be the third most common hr-HPV subtype causing adenocarcinoma, as well as missing information about obstetric outcomes after LLETZ.

Therefore, cone length should be carefully determined, especially in women with a desire to get pregnant, and always in accordance with the individual patient’s choice, and special awareness should be raised to balance the risk of adverse pregnancy events with oncologic safety. 

## 5. Conclusions

The decision about excision length should be discussed with the patient in terms of oncological safety and the risk of adverse pregnancy events. In addition, HPV 18 positivity is related to a higher rate of positive endocervical margins irrespective of TZ. An excision length >10 mm increases the number of cases with cervical tissue removed without further oncological benefit, especially in women with a visible TZ and HPV16/hr infection, which should be taking into account by clinicians during patient counseling, particularly with women wishing to conceive. 

## Figures and Tables

**Table 1:** Basic patient characteristics.

LLETZ (n = 854)			Excision Length ≤ 10 mm (n = 441)	Excision Length > 10 mm (n = 413)	*p*-Value
Age, years ^a^		36 (31–44)	34 (30–41)	38 (32–46)	*<0.001*
Involved endocervical margin ᵇ		147 (17.2)	85 (19.3)	62 (15.0)	0.099
Clear endocervical margin ᵇ		707 (82.8)	356 (80.7)	351 (85.0)	
Excision length, mm ^a^		10 (7–15)	8 (7–9)	15 (13–18)	*<0.001*
HPV 16/hr ᵇ		763 (89.3)	400 (90.7)	363 (87.9)	0.184
HPV 18 ᵇ		91 (10.7)	41 (9.3)	50 (12.2)	
Visible TZ ᵇ		701 (82.1)	375 (85.0)	326 (78.9)	*0.020*
Non-visible TZ ᵇ		153 (17.9)	66 (15.0)	87 (21.1)	
Histological diagnosis of LLETZ ᵇ	LSIL	124 (14.5)	74 (16.8)	50 (12.1)	0.053
	HSIL	673 (78.8)	339 (76.9)	334 (80.9)	0.153
	AIS	24 (2.8)	14 (3.2)	10 (2.4)	0.506
	SCC	31 (3.6)	14 (3.2)	17 (4.1)	0.562
	AC	2 (0.2)	0	2 (0.5)	0.143

Data are presented as ^a^ median (interquartile range) or ᵇ number (frequencies). Significant *p*-values are provided in italics. Abbreviations used are as follows: LLETZ, large loop excision of the transformation zone; TZ, transformation zone; HPV, human papillomavirus; LSIL low-grade intraepithelial lesion; HSIL, high-grade intraepithelial lesion; AIS, adenocarcinoma in situ; SCC, squamous cell carcinoma; AC, adenocarcinoma.

**Table 2:** Odds ratio for obtaining a negative endocervical margin as a function of the cone length > 10 mm according to HPV subtype.

	Endocervical Margin Status	Involved	Clear	OR	CI	*p* Value
visible TZ	HPV 16/hr					
	Excision length ≤ 10 mm ᵇ	59 (17.3)	282 (82.7)	Ref.	-	*0.027*
	Excision length > 10 mm ᵇ	32 (11.1)	257 (88.9)	1.68	1.06–2.67	
	HPV 18					
	Excision length ≤ 10 mm ᵇ	9 (26.5)	25 (73.5)	Ref.	-	0.170
	Excision length > 10 mm ᵇ	5 (13.5)	32 (86.5)	2.30	0.69–7.74	
non-visible TZ	HPV 16/hr					
	Excision length ≤ 10 mm ᵇ	18 (30.5)	41 (69.5)	Ref.	-	0.582
	Excision length > 10 mm ᵇ	19 (25.7)	55 (74.3)	1.24	0.58–2.68	
	HPV 18					
	Excision length ≤ 10 mm ᵇ	2 (28.6)	5 (71.4)	Ref.	-	0.787
	Excision length > 10 mm ᵇ	3 (23.1)	10 (76.9)	1.33	0.17–10.74	

Data are presented as ᵇ number (frequencies). Significant *p*-values are provided in italics. Abbreviations used are as follows: OR, odds ratio; CI, confidence interval; TZ, transformation zone; HPV, human papillomavirus.

## Data Availability

The data presented in this study are available on request from the corresponding author.
